# Early Detection of Type 1 Diabetes in First-Degree Relatives in Saudi Arabia (VISION-T1D): Protocol for a Pilot Implementation Study

**DOI:** 10.2196/70575

**Published:** 2025-04-14

**Authors:** Iman S Algadi, Yazed AlRuthia, Muhammad H Mujammami, Khaled Hani Aburisheh, Metib Alotaibi, Sharifah Al Issa, Amal A Al-Saif, David Seftel, Cheng-Ting Tsai, Reem A Al Khalifah

**Affiliations:** 1 Department of Pediatrics College of Medicine King Saud University Riyadh Saudi Arabia; 2 University Diabetes Centre King Saud Univeristy Medical City Riyadh Saudi Arabia; 3 Department of Clinical Pharmacy Pharmacy College King Saud Medical City Riyadh Saudi Arabia; 4 Department of Medicine College of Medicine King Saud University Riyadh Saudi Arabia; 5 College of Psychology Imam Mohammad ibn Saud Islamic University Riyadh Saudi Arabia; 6 Enable Biosciences South San Francisco, CA United States

**Keywords:** islet autoimmunity, type 1 diabetes mellitus, T1D, screening program, antibody detection by agglutination–polymerase chain reaction, PCR, ADAP, human leukocyte antigen, genetic risk score

## Abstract

**Background:**

Type 1 diabetes (T1D) is a growing global health concern, with a notable rise in incidence in Saudi Arabia. Despite the potential benefits of early detection through screening programs, such initiatives are currently lacking in Saudi Arabia and other Arab countries.

**Objective:**

This study aims to evaluate the feasibility, acceptability, and cost-effectiveness of a T1D-screening program targeting high-risk individuals, specifically children with a first-degree relative diagnosed with T1D.

**Methods:**

The VISION-T1D program is a prospective cohort study focused on the early detection of presymptomatic T1D by screening children aged 2-18 years. The primary screening method involves testing for islet autoantibodies, including insulin autoantibodies, glutamic acid decarboxylase autoantibodies, insulinoma associated-2 autoantibodies, and zinc transporter-8 autoantibodies. Optional genetic testing, including human leukocyte antigen phenotyping and the genetic risk score, is offered. Outcomes include the feasibility of the screening process, prevalence of early-stage T1D, psychological impacts, educational intervention effectiveness, progression rates to stage-3 T1D, and economic viability.

**Results:**

The VISION-T1D program began in May 2024. As of December 2024, a total of 176 families have been enrolled. Data collection will continue until April 2025, with final data analysis projected for mid-2025.

**Conclusions:**

The VISION-T1D study provides a practical approach to T1D screening tailored to the health care landscape of Saudi Arabia. The insights gained from this pilot program will inform the development of a national, population-based screening initiative designed to reduce diabetic ketoacidosis at diagnosis, improve long-term outcomes, and alleviate the economic burden of T1D. The VISION-T1D initiative could also serve as a scalable and sustainable model that can be adopted internationally, contributing to global efforts to manage and prevent T1D.

**Trial Registration:**

ClinicalTrials.gov NCT06513247; https://clinicaltrials.gov/study/NCT06513247

**International Registered Report Identifier (IRRID):**

DERR1-10.2196/70575

## Introduction

### Background

The incidence of type 1 diabetes (T1D) is rising globally, with Saudi Arabia reporting a surge to 31.4 per 100,000 youths annually, making a nearly 9-fold increase over the past decade [1,2]. This trend is compounded by high rates of diabetic ketoacidosis (DKA) at diagnosis [3], ranging from 40% to 77%, the highest in the Arab region [4,5]. DKA at T1D onset is associated with significant morbidity, mortality, and long-term complications [6-9]. DKA also imposes a substantial economic burden through direct and indirect medical costs [10-12]. Early detection by T1D screening is crucial in reducing DKA incidence and mitigating the health and economic impacts [13,14].

T1D pathogenesis involves a complex interplay of genetic predisposition and environmental factors, resulting in the autoimmune destruction of pancreatic β-cells [15,16]. Individuals with a first-degree relative (FDR) affected with T1D have a 15-fold higher lifetime risk of developing T1D compared to the general population [13]. Screening for islet autoantibodies identifies presymptomatic stages (stages 1 and 2) at least in children, adolescents, and young people, enabling timely interventions that may alter disease progression [13,17-19]. Individuals with multiple positive islet autoantibodies have a 99% lifetime risk of progressing to clinical stage-3 T1D, with 84% developing insulin-dependent diabetes within 15 years of seroconversion [18-20]. Screening programs in several countries have successfully reduced DKA at diagnosis, improved long-term outcomes, and lowered health care costs [13,14].

Despite successful screening initiatives in other countries, Saudi Arabia and neighboring Arab countries lack such programs. Moreover, most prior efforts have been confined to research settings, leaving a gap in knowledge about real-world implementation in health care settings [14,18,20,21]. The VISION-T1D protocol (NCT06513247) aims to bridge this gap by evaluating the feasibility and cost-effectiveness of a pilot, T1D-screening program for FDRs of individuals with T1D in Saudi Arabia. Aligned with Saudi Arabia’s National 2030 Vision, which prioritizes health improvement and the reduction of chronic disease burden, the VISION-T1D initiative aims to establish a robust framework for broader, population-based, T1D-screening implementation in Saudi Arabia. Furthermore, it could also serve as a scalable and sustainable model for international adoption, thereby contributing to global efforts to manage and prevent T1D.

### Study Objectives

#### Primary Objectives

The primary objectives are as follows:

- Evaluate the feasibility of implementing a pilot, T1D-screening program for early detection in FDRs of patients with T1D in Saudi Arabia.
- Determine the prevalence of stages-1, -2, and -3 T1D among FDRs of patients with TID in Saudi Arabia.

#### Secondary Objectives

The secondary objectives are as follows:

- Examine the psychological impacts of T1D screening on caregivers, including changes in anxiety, depression, distress level, and health-related quality of life before and after receiving screening results.
- Evaluate the effect of early detection on reducing DKA incidence at stage-3 T1D onset.
- Analyze the cost-effectiveness of implementing a T1D-screening program in Saudi Arabia.
- Evaluate the natural history of T1D progression in identified presymptomatic individuals, tracking time to clinical onset and changes in health outcomes over time.
- Examine the effectiveness of educational interventions on caregivers’ knowledge of T1D screening and early identification.
- Investigate the genetic predisposition to T1D within our population through human leukocyte antigen (HLA) testing and validate the genetic risk score (GRS) 2 in the Saudi population.

## Methods

### Study Design and Setting

The Vision-T1D study is a prospective cohort designed to screen children aged 2-18 years who have an FDR with T1D (index case). Participants are recruited from the University Diabetes Centre (UDC) at King Saud University Medical City (KSUMC) in Riyadh, Saudi Arabia. The diagnosis of T1D in index cases is based on the American Diabetes Association criteria [22], and T1D classification is determined by a clinical diagnosis from a specialized endocrinologist, with or without confirmatory islet autoantibodies testing. Exclusion criteria included index cases diagnosed with other forms of diabetes and non-Saudi children due to different ethnic and genetic backgrounds.

### Ethical Considerations

The study was approved by the Institutional Review Board of King Saud University Ethics Committee (approval: E-248566). The pilot phase began in May 2024, with 1 year dedicated to recruitment and screening. All procedures were conducted in accordance with the ethical standards of the Declaration of Helsinki and national research ethics guidelines.

Informed consent was obtained from all participants or their legal guardians prior to data collection. Privacy and confidentiality were maintained according to GCP standards. Participants identified as at risk or presymptomatic for T1D will enter a 4-year clinical follow-up phase, culminating in a total study duration of 5 years.

Participants did not receive financial compensation for participation. However, We have received funding for the project as described in the acknowledgment section.

### Study Process and Interventions

**Figure 1:**
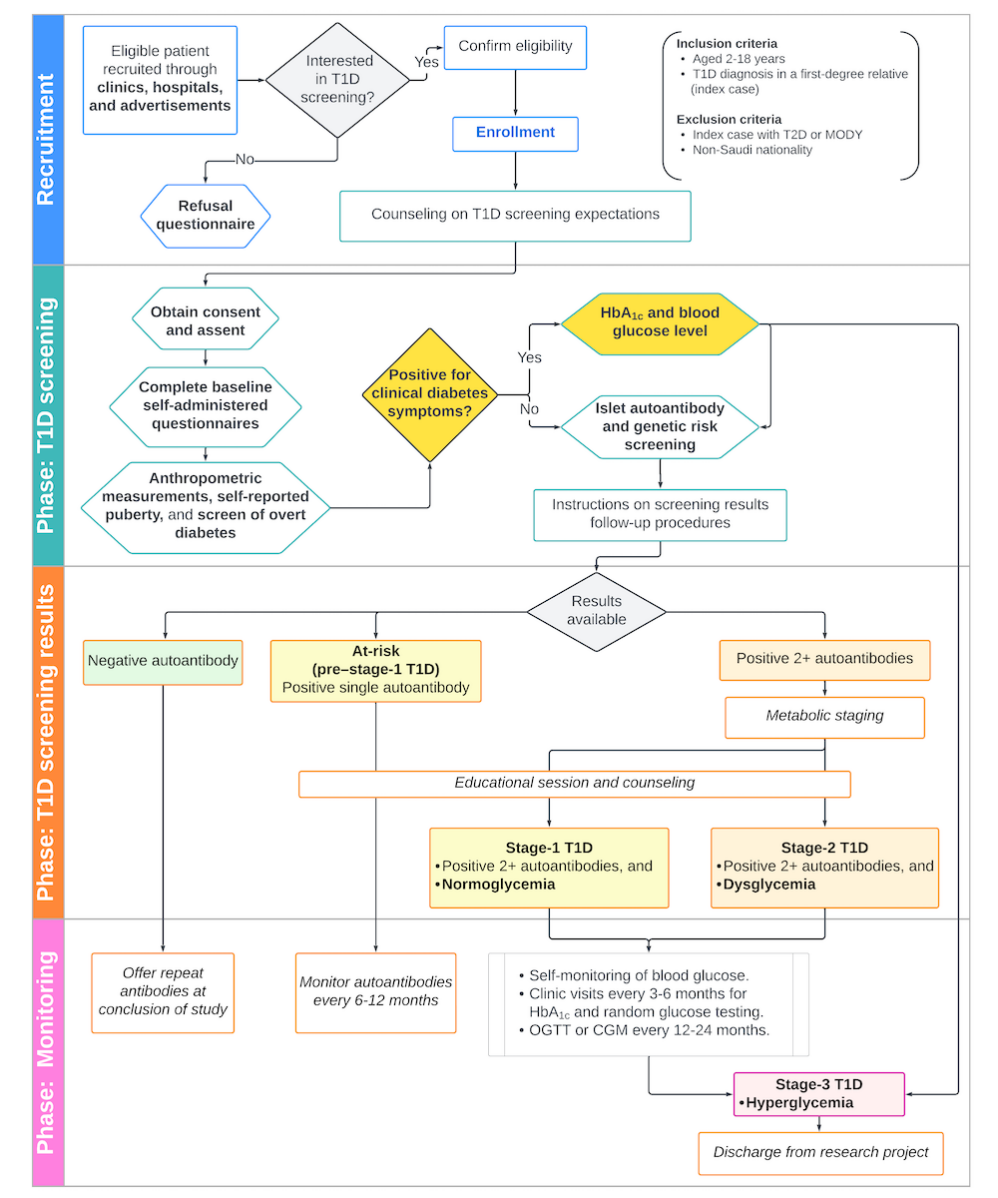
The study flowchart. CGM: continuous glucose monitoring; HbA_1c_: hemoglobin A_1c_; MODY: maturity-onset diabetes of the young; OGTT: oral glucose tolerance test; T1D: type 1 diabetes; T2D: type 2 diabetes.

The VISION-T1D study consists of 3 well-structured phases: screening, results communication and counseling, and long-term clinical follow-up. Each phase is designed to provide tailored interventions, ensuring appropriate care, monitoring, and education for children and their families (Figure 1).

#### Phase 1: T1D Screening

Families are recruited and scheduled for an enrollment and screening visit, where they receive comprehensive counseling about participation and education on T1D screening. Upon obtaining informed consent and assent, participants undergo baseline assessments, including completion of questionnaires and the collection of blood samples for islet autoantibody testing. Optional genetic screening for HLA phenotyping and GRS analysis is also offered to evaluate genetic predisposition to T1D. To enhance recruitment, a home testing option is provided, allowing families to conveniently perform finger-prick testing and data collection at home.

#### Phase 2: Results Communication, Education, and Metabolic Staging

After screening results are available, a research physician reviews the findings. Participants who test positive for islet autoantibodies are invited to a follow-up visit that includes personalized counseling, targeted educational interventions, and additional metabolic staging to determine their T1D stage. Participants with negative islet autoantibody results are notified by the research staff and are invited for repeat autoantibody screening at the conclusion of the long-term follow-up period (approximately 4-5 years later).

#### Phase 3: Long-Term Clinical Follow-Up

Participants identified as having presymptomatic T1D (stage 1 or 2) or those at risk based on autoantibody markers enter a 4-year clinical follow-up phase. During this period, regular monitoring of disease progression is conducted through metabolic assessments and psychological evaluations. Participants receive continuous support tailored to their risk level and diagnosis.

This systematic, 3-phase approach ensures that participants receive appropriate care, counseling, and monitoring based on their screening outcomes.

### Phase 1: T1D-Screening Procedures and Data Collection

#### Recruitment and Enrollment

Active and passive recruitment strategies are used. Research staff identified potential participants by reviewing patient lists from KSUMC diabetes clinics and pediatric endocrinology inpatient records. Recruitment materials, including advertisements with QR codes, are shared via the UDC and on social media platforms. The QR codes were linked to an introductory video explaining the study and eligibility criteria, along with a contact submission form. Families who decline participation are asked about the reason for refusal.

#### Informed Consent

Eligible families are scheduled for T1D-screening visits, where informed consent and assent are obtained. A comprehensive educational approach was developed to ensure participants receive standardized information and fully understand the T1D-screening process. This included an animated video and an educational booklet. The video, in Arabic, explains the screening program and key concepts, such as T1D pathophysiology, its stages, and clinical presentation. It emphasizes the benefits of early detection in preventing DKA and improving long-term outcomes; outlines the screening process; and provides an overview of current and emerging T1D treatments, including insulin therapy and disease-modifying interventions. The educational booklet complemented the video, using infographics to illustrate T1D pathophysiology, stages, symptoms, and benefits of screening. It also outlined study expectations and provided key study contact information.

#### Intervention: T1D Screening

##### Islet Autoantibodies Screening

The program uses antibody detection by agglutination–polymerase chain reaction (ADAP) assay, a cutting-edge technology developed by Enable Biosciences. The assay measures islet autoantibodies [23-27], including insulin autoantibodies, glutamic acid decarboxylase autoantibodies, IA-2 autoantibodies, and zinc transporter 8 autoantibodies, using dried blood spots on filter paper. The ADAP assay offers notable advantages over other assays for large-scale analysis, such as cost-effectiveness, high sensitivity and specificity, and minimal sample volume requirements. These features make it suitable for large-scale applications, including home testing [25-27].

##### Genetic Risk Screening

This pilot study integrated optional HLA phenotyping and GRS testing to deepen the understanding of T1D pathogenesis in the Saudi population. Analyses will be conducted by Enable Biosciences, the Barbara Davis Center for Childhood Diabetes, and delegates [28,29]. These tests are used for research purposes only.

###### HLA Phenotyping

Testing focuses on identifying high-risk HLA genotypes associated with T1D, particularly variants in the HLA region (MHC class II alleles), which account for over 50% of T1D heritability [13]. This approach aims to provide insights into T1D risk associations in the Saudi population, especially in participants with negative islet autoantibodies. HLA typing is performed using oligonucleotide probes for *HLA-DRB1*, *DQA1*, and *DQB1* alleles as previously described [30].

###### GRS Testing

The GRS aggregates the cumulative contribution of multiple genetic variants into a single score estimating an individual’s genetic predisposition to T1D [29,31-33]. This study uses GRS2, an advanced version that incorporates HLA haplotypes, non-HLA loci, and interactions between HLA variants [34]. Testing is conducted using a Kompetitive allele-specific polymerase chain reaction (PCR) assay, as described earlier [35,36]. The Kompetitive allele-specific PCR assay is a cost-effective and efficient alternative to the traditional DNA array and imputation-based methods. It successfully genotypes 60 of the 67 single nucleotide polymorphisms in GRS2, with the remaining single nucleotide polymorphisms excluded due to genotyping challenges. This study represents one of the first applications of GRS2 in FDRs of patients with T1D and the first validation of its use within the Saudi population.

##### Specimen Collection

Blood samples are collected via fingerstick or venous sampling onto dried blood spot filter paper, based on participant preference. For families opting for home testing, parents collect sample cards and detailed instructions from the clinic, perform the testing at home, and return the samples for processing. All collected samples are stored at –80 °C before being batched and shipped to the United States for analysis.

#### Measures

##### Demographics and Clinical Characteristics

Families complete a self-administered questionnaire upon enrollment to collect baseline demographics and clinical characteristics. Information includes the age, sex, medical history, and current medications of the participating child, as well as family demographics such as parental age, marital status, number of siblings, education levels, family income, residential region, and family medical history focused on diabetes or autoimmune diseases among FDR and any self-reported parental comorbidities. For the index case with T1D, the questionnaire captures clinical details, including the relationship to the participant, age at diagnosis, current age, insulin regimen, history of DKA at onset or recurrent DKA, and diabetes-related complications. If multiple FDRs have T1D, similar information is collected for each.

##### Clinical T1D Screening

Participants are screened for overt diabetes symptoms, such as polyuria, polydipsia, polyphagia, and unexplained weight loss. Research staff conduct point-of-care hemoglobin A_1c_ (HbA_1c_) and random blood glucose tests if any symptoms are reported. Participants with abnormal blood sugar levels are referred to a research physician for further evaluation, which may include an oral glucose tolerance test (OGTT) and counseling. Those meeting the diagnostic criteria for stage 3 T1D will receive standard medical care and education for new-onset diabetes.

##### Health Literacy

The Single-Item Literacy Screener tool evaluates parental health literacy using a 5-point Likert scale. A score of 4 or higher indicates adequate health literacy, while a score of 3 or lower suggests limited literacy [37-39].

##### T1D-Screening Acceptability Assessment Scale

This self-administered tool evaluates caregivers’ acceptability of the T1D-screening program through 10 items rated on a 5-point Likert scale. Domains include affective attitude, the ethicality of the screening, perceived effectiveness, self-efficacy, opportunity costs, communication, burden, and general and cultural acceptability. Total scores range from 10 to 50, with higher scores indicating greater acceptability [40].

##### Psychological Measures

A set of validated Arabic tools assess caregiver stress, anxiety, depression, and quality of life at baseline, after results disclosure, and during long-term follow-up.

###### Anxiety Symptoms

The Generalized Anxiety Disorder 7-item scale screens for generalized anxiety over the past 2 weeks using a 4-point Likert scale. A score of 8 or more indicates possible generalized anxiety disorder, with 92% sensitivity and 76% specificity [40,41].

###### Depressive Symptoms

The Patient Health Questionnaire-9 screens for depression. A score of more than 10 suggests possible major depressive disorder, with a sensitivity and specificity of 88% [42-44]. Research staff will screen responses indicating suicidal ideation and prompt immediate referral to emergency care.

###### Health-Related Quality of Life

The EQ-5D-5L tool from the EuroQol Group measures health across 5 dimensions—mobility, self-care, usual activities, pain or discomfort, and anxiety or depression—each rated on 5 levels. The EuroQol Visual Analogue Scale allows participants to rate their overall health from 0=worst to 100=best [45]. The results are scored using a value set specific to Saudi Arabia, providing a comprehensive quality of life score [46].

###### Distress

The Subjective Units of Distress Scale measures anxiety and discomfort on a 0-100 scale, with higher scores indicating greater distress. The Subjective Units of Distress Scale is widely used and validated in clinical settings for assessment and monitoring of changes over time [47-49].

##### Anthropometric Measurements

Baseline assessments will include weight, height, BMI, and self- or parental-reported pubertal status [50].

### Phase 2: Result Disclosure, Education, and Metabolic Staging

#### Results Disclosure

Participants receive written reports explaining the results, estimated risk of progression, long-term follow-up recommendations, and key contact information. The process of sharing results varies based on screening outcomes:

##### Participants With Negative Results

Families of participants who test negative for islet autoantibodies are contacted by the research staff to receive their results report. They are instructed to contact the research team if T1D develops in the future and are invited for repeat antibody testing at the end of the follow-up phase.

##### Participants With Positive Results

Families of participants who test positive for multiple islet autoantibodies are invited to a one-to-one counseling visit with a research physician to discuss the result, followed by a group education and metabolic staging to determine their T1D stage.

##### Participants With Equivocal Results

Families of participants with a single autoantibody are invited for a one-to-one counseling session with a physician to discuss the result. They then join the same group educational session as those with positive results.

#### Genetic Testing Disclosure

Genetic testing results are shared with families only upon request to avoid unnecessary alarm given the elevated baseline risk among FDRs and the lack of validated Saudi population–based genetic risk estimation. When shared, these results are presented in a dedicated educational session designed to help families understand the complexities of genetic predisposition to T1D.

#### Educational Curriculum for Participants With Positive or Equivocal Results

The educational curriculum was developed based on a comprehensive robust educational framework and expertise [51]. It is delivered in small group sessions by experienced endocrinologists, diabetes educators, and dietitians. The curriculum covers key topics such as T1D pathophysiology, disease progression stages, and the importance of early detection. Participants learn to identify their specific stage and risk of progression and recognize factors that influence clinical diabetes onset. The curriculum highlights insulin’s role as the primary treatment for clinical T1D (stage 3), addressing common concerns and misconceptions. Participants are also trained to monitor their condition by recognizing early T1D symptoms, performing blood glucose checks, interpreting results, and determining when insulin therapy or medical advice is needed.

The program underscores nonpharmacological strategies for reducing the risk of T1D progression through healthy eating and lifestyle modifications and provides an overview of current and future T1D research. The psychological and emotional aspects of receiving T1D screening results are also addressed, and access to support is provided.

#### Psychological Support

The psychological and emotional impact of T1D risk disclosure is carefully addressed throughout the process. Families experiencing significant psychological burdens are offered counseling sessions and access to professional psychological support. Additionally, all families can self-refer to these services as needed. These measures are designed to alleviate stress, promote resilience, and ensure families feel supported during the follow-up period.

#### Metabolic Staging

Participants with multiple positive islet autoantibodies undergo metabolic staging to determine their T1D stage. This includes HbA_1c_ measurement, fasting glucose, 2-hour OGTT, or continuous glucose measurement (CGM) if OGTT is not feasible.

##### OGTT Measurement

Performed after an overnight fast of at least 8 hours. Participants consume a glucose solution dosed at 1.75 g/kg (up to 75 g). Serum glucose levels are measured at fasting baseline and 120 minutes after ingestion.

##### HbA_1c_ Measurement

Serum samples will be processed in the laboratory per American Diabetes Association–recommended standards.

##### CGM Measurement

Used as an alternative for participants unable or unwilling to complete an OGTT. CGM glucose profiles over a 2-week period will be reviewed for staging.

### Phase 3: Long-Term Monitoring and Clinical Follow-Up

#### Monitoring for Disease Progression

Participants with positive single or multiple autoantibodies will enter a 4-year follow-up phase. During this period, regular assessments are performed to monitor T1D disease progression, track time to clinical onset, and observe any changes in metabolic staging. Monitoring intervals vary by stage and risk level [18,20]. Participants progressing to stage-3 T1D will transition to standard care.

##### Self-Monitoring of Blood Glucose

All participants with multiple positive islet autoantibodies results will be instructed to perform self-monitoring of blood glucose testing at home twice over a 2-week period after receiving their results, including one fasting test and one 2-hour postprandial test after a heavy meal. This testing should be repeated every 1-3 months and during any illness. Participants will be provided with contact information and guidance on when to seek medical advice.

##### Stage-1 Monitoring

Participants categorized as stage-1 T1D (positive autoantibodies with normal glycemia) will have clinic visits every 6 months for HbA_1c_ and random glucose testing. An OGTT or CGM will be performed every 24 months or earlier if there is a 10% or more rise in HbA_1c_ from baseline.

##### Stage-2 Monitoring

Participants categorized as stage-2 T1D (positive autoantibodies with dysglycemia) will have more frequent clinic visits every 3 months for HbA_1c_ and glucose testing. An OGTT or CGM will be performed annually or sooner if there is a 10% or more rise in HbA_1c_ from baseline.

##### Monitoring for Single Autoantibody Results

Participants with a single autoantibody undergo metabolic assessment only if symptoms develop. For children younger than the age of 6 years, autoantibody screening will be repeated within 6 months for confirmation, then annually throughout the study. For children aged 6 years or older, annual screening will be conducted for the duration of the study. Autoantibody monitoring will be discontinued if a participant seroconverts to a negative status. No physician visits are required, and families are encouraged to contact the research team if T1D symptoms arise in the future.

##### Monitoring for Participants With Negative Autoantibodies

All participants who test negative for autoantibodies will undergo follow-up autoantibody testing at the conclusion of the 5-year study period.

#### Other Monitoring Measures

##### Clinical Measurements

Weight, height, and BMI are assessed annually for participants with positive autoantibodies.

##### Psychological Assessment

Parents of children with positive autoantibody results will undergo psychological assessment within one week of receiving screening results. Assessments are repeated during follow-up at 6 and 12 months to track changes over time.

### Outcome Definitions and Metrics

#### Feasibility Metrics

The feasibility of the screening program will be evaluated using the following key metrics.

-
  Recruitment rates: The proportion of eligible participants successfully recruited into the study.
  Screening refusal rates: The percentage of eligible participants who decline participation.
  Completed screening visit rates: The percentage of participants completing the initial screening visit (phase 1).
  Completed counseling visit rates: The percentage of participants with positive autoantibody results who complete the counseling session and educational curriculum (phase 2).
  Completion of long-term follow-up: The percentage of participants with presymptomatic T1D who complete their monitoring visits during the 4-year follow-up period (phase 3). Participants progressing to clinical T1D before the study conclusion will transition to standard clinical care and be considered as having completed follow-up.

#### Prevalence of Presymptomatic T1D at Screening

Metabolic staging performed during the screening results visit (phase 2) will classify participants into the following stages.

-
  Stage-1 T1D: Presence of two or more islet autoantibodies with normal glycemia. Criteria include HbA_1c_ <5.7%, fasting blood glucose <100 mg/dL, and a 2-hour OGTT <140 mg/dL. For CGM users, values >140 mg/dL should be less than 10% of the time over 10 days of continuous wear.
  Stage-2 T1D: Presence of two or more islet autoantibodies with dysglycemia. Criteria include HbA_1c_ between 5.7% and 6.4%, fasting blood glucose between 100-125 mg/dL, and a 2-hour OGTT between 140-199 mg/dL. For CGM users, values more than 140 mg/dL should be between 10% to less than 20% of the time over 10 days of continuous wear.
  Stage-3 T1D: Clinical onset of T1D marked by one or more islet autoantibodies and hyperglycemia. Criteria include HbA_1c_ 6.5%, fasting blood glucose levels 126 mg/dL, and a 2-hour OGTT 200 mg/dL. For CGM users, values >140 mg/dL must occur at least 20% of the time over 10 days of continuous wear.
  At risk (pre–stage-1 T1D): Defined as the presence of a single islet autoantibody with normal glycemia.
  Isolated genetic predisposition: Defined as the presence of positive genetic risk markers without accompanying islet autoantibodies.

#### Incidence of DKA

The incidence of DKA at the clinical onset of T1D will be tracked during the long-term follow-up phase (phase 3). DKA occurrences will be captured from clinical records and follow-up visits.

#### T1D Progression Rate

The annual progression rate to stage-3 T1D will be tracked in participants testing positive during the follow-up phase (phase 3) and categorized based on their initial stage.

#### Effectiveness of Educational Interventions

The effectiveness of the educational curriculum will be evaluated using pre- and postintervention tests completed by caregivers. These tests are developed to measure changes in knowledge related to the curriculum’s objectives, providing a comprehensive assessment of the intervention's effectiveness in preparing caregivers for presymptomatic T1D management.

#### Health Care Resource Use for Cost-Effectiveness Analysis

Health care resource use will include both program-related and external health care resources. Program-related costs will encompass autoantibody testing, metabolic profiling, results notification visits, and educational or follow-up visits. Non–program-related costs will include diabetes clinic visits (eg, consultations with physicians, educators, nutritionists, or psychologists), emergency department visits for diabetes or DKA, hospitalizations for diabetes, laboratory and imaging studies, and prescription medications for diabetes management either inpatient or outpatient.

### Statistical Analyses

#### Sample Size

The target enrollment is 1300 participants (or 322 families), based on the feasibility of recruiting 70% of eligible families from the UDC clinic cohort. The estimated seropositivity rate for islet autoantibodies among children with an FDR diagnosed with T1D is estimated at 5% to 15%, with a 90% significance level and 80% power. Considering that the UDC serves approximately 450-500 children with T1D, and that the typical Saudi family has 4-6 children younger than the age of 18 years, this sample size is calculated to be sufficient. Recruitment will be expanded to FDRs outside the UDC cohort if the family target is met without reaching the participant’s goal.

#### Statistical Analysis

Descriptive statistics will report continuous data as mean (SD) for normally distributed variables and categorical data as frequency and percentage (N, %). Feasibility metrics and T1D stages will also be summarized as percentages.

To evaluate the psychological effects of T1D screening on caregivers, pre- and postscreening levels of anxiety, depression, and distress will be compared using paired 2-tailed *t* tests or Wilcoxon signed-rank tests, depending on data distribution. The effectiveness of educational counseling will be analyzed similarly, comparing pre- and postintervention test scores. Longitudinal changes in the psychological measures will be evaluated using repeated-measures ANOVA or mixed effects models to track patterns over time.

For participants with presymptomatic T1D, progression rates to stage 3 will be analyzed using Kaplan-Meier survival analysis. Time to event will be calculated from screening age to age at stage-3 T1D diagnosis or last follow-up. Cox proportional hazards models will assess factors associated with disease progression, such as baseline autoantibody titers, genetic risk scores, and demographic variables.

At the 1-year follow-up, cost-effectiveness will be assessed by reporting total costs for all screened participants, cost per child screened, and cost per case detected. The incremental cost-effectiveness ratio calculations will compare outcomes for screened children with controls diagnosed with stage-3 T1D in the general population. The incremental cost-effectiveness ratio will incorporate potential benefits, such as reduced DKA incidence and improved HbA_1c_ levels, while adjusting for control characteristics like age and FDR status.

## Results

The VISION-T1D pilot screening program was funded and launched in May 2024, with the initial screening phase scheduled to conclude in April 2025. As of November 2024, a total of 176 eligible families had enrolled, representing approximately 39.1% of the target sample size of 322 families. Final data analysis for the initial screening phase is projected for mid-2025.

## Discussion

The VISION-T1D protocol outlines a comprehensive approach to assess the feasibility and impact of a T1D screening among FDRs of individuals with T1D in Saudi Arabia. This model is tailored to address the unique demographic, cultural, and health care characteristics of the Arab region, aiming to deepen understanding of the prevalence and patterns of autoimmunity in at-risk populations. The data generated will provide valuable epidemiological insights to inform future health care policies and targeted interventions.

A key strength of this study is its focus on translating screening programs from the research setting to practical, real-world implementation. It provides a detailed roadmap for integrating T1D screening into health care systems, addressing scientific and operational complexities, particularly within the Arab region. The use of culturally sensitive consent materials, diverse educational methods, and clear protocols ensures that participants and caregivers are well informed, supported, and empowered to manage their health proactively. Additionally, the adoption of the ADAP assay—a cost-effective, sensitive, and low-sample-volume technology—enhances scalability and operational efficiency, making the program accessible for large-scale applications.

This study addresses significant unmet needs in T1D care and research in the Middle East, offering critical insights into genetic predispositions and disease progression in the Saudi population. One of the program’s pivotal contributions is the early identification of candidates for T1D prevention therapies. Early detection through screening will facilitate timely intervention with disease-modifying therapies that can delay or prevent the onset of clinical T1D. This proactive approach aligns with global health strategies advocating for prevention and early intervention to mitigate the long-term burden of chronic diseases. Furthermore, this initiative lays the groundwork for the future of prevention trials in Saudi Arabia, contributing to the development of a sustainable infrastructure for T1D research and management.

The study also tackles logistical challenges, such as recruitment, sample analysis, and generalizability, through a combination of recruitment strategies, robust educational tools, and systematic follow-up procedures. While reliance on international laboratories for sample analysis introduces potential delays, the program is designed with long-term sustainability in mind, including plans to establish a centralized laboratory for the ADAP assay in Saudi Arabia.

Despite its strengths, the study has limitations. Focusing on FDRs may restrict generalizability to the broader population, as prior exposure to T1D within the family could influence the acceptability of the screening program. Additionally, cultural and social factors specific to Saudi Arabia may shape perceptions and behaviors related to T1D screening and management, potentially affecting intervention outcomes. Furthermore, the relatively short follow-up period, constrained by funding, limits the ability to assess long-term outcomes and the program’s sustainability. Extended follow-up would provide a deeper understanding of the enduring effects of early detection and educational interventions on disease progression and management. Future extensions of this study are based on funding availability or collaborations with national screening programs may allow for prolonged follow-up.

In conclusion, this study represents a critical advancement in addressing the challenges of T1D screening and management of presymptomatic T1D. By integrating innovative screening technology with a robust educational framework and systematic follow-up, this program demonstrates the potential to enhance health outcomes while offering a scalable and sustainable approach to managing presymptomatic T1D. The outcomes garnered from this research will provide a foundation for implementing practical T1D-screening programs and establishing a framework adaptable to similar initiatives in neighboring Arab countries. By addressing both scientific and operational challenges, this study serves as a valuable resource for policy makers, health care providers, and researchers striving to integrate effective T1D management strategies into routine health care practice, ultimately improving the quality of care for at-risk populations.

## Abbreviations

ADAP: antibody detection by agglutination–polymerase chain reaction
CGM: continuous glucose monitoring
DKA: diabetic ketoacidosis
FDR: first-degree relative
GRS: genetic risk score
HbA_1c_: hemoglobin A_1c_
HLA: human leukocyte antigen
KSUMC: King Saud University Medical City
OGTT: oral glucose tolerance test
PCR: polymerase chain reaction
T1D: type 1 diabetes
UDC: University Diabetes Centre
